# Ammonia-oxidising bacteria not archaea dominate nitrification activity in semi-arid agricultural soil

**DOI:** 10.1038/srep11146

**Published:** 2015-06-08

**Authors:** Natasha C. Banning, Linda D. Maccarone, Louise M. Fisk, Daniel V. Murphy

**Affiliations:** 1 Soil Biology and Molecular Ecology Group, School of Earth and Environment, Institute of Agriculture, The University of Western Australia, Crawley, WA 6009, Australia

## Abstract

Ammonia-oxidising archaea (AOA) and bacteria (AOB) are responsible for the rate limiting step in nitrification; a key nitrogen (N) loss pathway in agricultural systems. Dominance of AOA relative to AOB in the *amoA* gene pool has been reported in many ecosystems, although their relative contributions to nitrification activity are less clear. Here we examined the distribution of AOA and AOB with depth in semi-arid agricultural soils in which soil organic matter content or pH had been altered, and related their distribution to gross nitrification rates. Soil depth had a significant effect on gene abundances, irrespective of management history. Contrary to reports of AOA dominance in soils elsewhere, AOA gene copy numbers were four-fold lower than AOB in the surface (0–10 cm). AOA gene abundance increased with depth while AOB decreased, and sub-soil abundances were approximately equal (10–90 cm). The depth profile of total archaea did not mirror that of AOA, indicating the likely presence of archaea without nitrification capacity in the surface. Gross nitrification rates declined significantly with depth and were positively correlated to AOB but negatively correlated to AOA gene abundances. We conclude that AOB are most likely responsible for regulating nitrification in these semi-arid soils.

Nitrification, the microbially-mediated process which converts ammonia to nitrate (through nitrite), is the major pathway by which nitrogen (N) can be lost from terrestrial ecosystems. The autotrophic ammonia-oxidising bacteria (AOB), which produce the key functional enzyme ammonia monooxygenase (AMO), have historically been thought solely responsible for most ammonia oxidation, the rate limiting step in nitrification, in terrestrial and aquatic ecosystems. This changed following the discovery of intact *amoA* genes in mesophilic Crenarchaeota, members of the domain Archaea^1^. Ammonia-oxidising archaea (AOA) have since been shown to numerically dominate AOB in several European agricultural and pristine soils^2^ and subsequently elsewhere^3^,^4^. More recent phylogenetic analyses have placed the AOA in the new phylum Thaumarchaeota^5^.

In soils globally, the availability of inorganic nitrogen (ammonium and nitrate) is important to plant nutrition and can regulate net primary productivity. In agricultural soils, nitrogen loss (facilitated by nitrification) can be substantial, decreasing the efficiency of nitrogen fertilizer use at huge economic cost^6^. Furthermore, nitrogen loss through nitrate leaching contributes to groundwater pollution and the conversion of nitrate to nitrous oxide (N_2_O) via denitrification pathways contributes to soil greenhouse gas emissions^7^. One major question yet to be answered is to what extent AOA are important in the nitrification process.

The presence of an *amoA* gene, transcript or protein is not sufficient to infer *in situ* ammonia oxidization activity^5^. Consequently, there is considerable uncertainty regarding the relative contributions of AOB and AOA to soil nitrification. Recently, there has been evidence that AOA functionally dominate in acidic soils (with pH < 5.5)^8^. Elsewhere, AOB have been shown to dominate nitrification activity, even where they were numerically less dominant than AOA^9^,^10^,^11^.

The relative importance of AOA versus AOB to nitrification in semi-arid agricultural soils also remains unclear. Semi-arid and arid lands constitute one-third of the global land area and are widely used for agricultural production^7^. In the semi-arid south western Australian grain-belt AOA have been found to be either similar to or less abundant than AOB, in surface soil horizons^12^,^13^. The region typically has acidic sandy soils with low organic matter content, where inorganic nitrogen fertilizers are required for crop production. As two of the main drivers thought to provide a competitive niche for AOA over AOB are a low soil pH^14^,^15^ and low substrate (i.e. ammonia) availability^16^, the low abundance of AOA in earlier studies was unexpected^17^. However, it has also been hypothesized that nitrogen supply through inorganic fertilizer application, as opposed to an organic nitrogen supply pathway, may favour AOB activity in agricultural soils^5^,^18^.

The impact of nitrogen fertilizer application as well as other agricultural practices (e.g. liming, organic matter amendment, tillage) is predominantly in the soil surface horizon and it has not been investigated whether the higher relative abundance of AOB persists below the surface soil layer or if the surface dominance of AOB is management induced. Soils exhibit strong environmental gradients with depth and little is known more widely about the distribution of AOA and AOB abundance and function down the soil profile. In many terrestrial ecosystems, microbial biomass and activity declines with increasing depth^19^,^20^. However, studies have found AOA abundance either stays relatively constant with soil depth or even increases with soil depth (this was observed in an analysis of total archaeal abundance in which most of the population was identified as Thaumarchaeota)^20^, while AOB abundance generally declines^2^,^10^.

As such, the aims of this study were to i) quantify the distribution of AOA and AOB in the profile of semi-arid soils, ii) examine the relationship between gross nitrification rates and *amoA* gene abundance of AOA and AOB, and iii) determine the influence of soil pH and soil organic matter on AOA and AOB populations. Two agricultural field trial sites with treatments specifically manipulating soil pH and organic matter were selected for sampling. Both sites have acidic, sandy soils typical of the broader grain-belt regions of southern Australia.

## Results and Discussion

**Figure 1:**
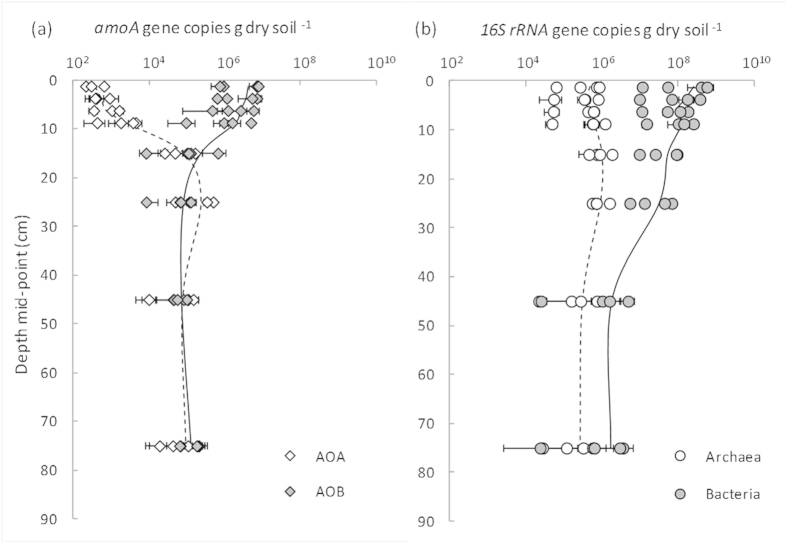
Abundance of bacterial and archaeal *amoA* genes (**a**) and bacterial and archaeal *16S rRNA* genes (**b**) in Western Australian semi-arid agricultural soils. Data points represent means of three soil cores (per soil layer) collected from trial sites where soil pH (−lime versus +lime) or soil organic matter (−OM versus +OM) had been historically altered. Error bars represent ± 1 SE. The mean of all treatments combined at each depth are shown by the dashed line (archaea) or solid line (bacteria). Note: gene copy numbers are plotted on a log_10_ scale.

Irrespective of agricultural management, there were distinct depth profiles of both AOA and AOB populations. AOB populations were significantly higher (*P* < 0.001) in the surface layer (0–10 cm) compared to the sub-soil (10–90 cm; Fig. 1a).This was consistent with the depth distribution of total bacterial population (Fig. 1b) and was expected given depth gradients in soil organic matter, nutrients and aeration. In contrast the AOA population was low in the surface layer but of similar magnitude to AOB below 10 cm (Fig. 1a). The low AOA population in the surface was not mirrored by the total archaeal population which varied little with depth (Fig. 1b). This suggests that in the surface soil horizon at all sites there is either (i) a population of AOA that is not detected with the primers used in this study or (ii) a large population of non-ammonia-oxidising archaea, such as the Euryarchaeota (which includes methanogens) or group 1.1c Thaumarchaea which have been found in many acidic soils but have no known link to *amoA* phylogeny^8^,^21^.

The primers used in this study, developed by Francis *et al.*^22^ for use in the marine environment, have been widely used for qPCR determination of AOA abundance in acidic agricultural soils^12^,^13^,^15^,^23^ and elsewhere^24^,^25^. The primers amplify a near full-length *amoA* gene product (635 bp) and thus *in silico* primer analysis is limited by the availability of full-length *amoA* sequences. Nonetheless, a limited *in silico* analysis of all available archaeal *amoA* sequences (n = 15; as published previously^26^) covering the primer target regions (with the exception of the first three bases of the forward primer which was only covered by the soil fosmid clone 54d9^1^), revealed between 0 and 2 mismatches with the forward primer and between 0 and 3 mismatches with the reverse primer. However, the majority of the mismatches were with AOA associated with, or isolated from, non-soil environments (marine, estuarine, hot spring or the sponge symbiont *Cenarchaeum symbiosum*). There were no mismatches with either primer for the sandy soil fosmid clone 54d9^1^ or the two *Nitrosospheara* sequences (belonging to “group 1.1b” which have been shown to be the dominant AOA in many soils^21^,^26^). Furthermore, none of the mismatches that were present were in the five bases at the 3’ end of the forward primer or the 5’ end of the reverse primer, the regions where target-primer matches are the most important for successful PCR amplification^27^. Thus, there was no evidence of AOA amplification being restricted by primer coverage limitations. However, geographic location on the continental scale has been purported to effect soil AOA population structure^26^ and it cannot be ruled out that these deeply weathered, ancient and geographically isolated Western Australian soils^28^ harbor novel AOA not detected by the current primers.

Nonetheless, we hypothesize that annual applications of inorganic nitrogen (20–100 kg N ha^−1^) have favoured AOB over AOA. Previous studies have indicated that AOA may have a competitive advantage at low ammonia concentrations due to their higher substrate affinity^29^ or possibly due to higher sensitivity to growth inhibition at high ammonia concentrations^16^. Activity of soil AOA is generally detected below 15 μg NH_4_^+^−N per g soil^5^, although this is not always the case^30^. The measured ammonium concentrations in this study were all low (<1 μg NH_4_^+^−N per g soil) at the time of sampling, and declined with depth. However, soil was collected in summer prior to cropping and this does not reflect the historical annual applications of urea and inorganic ammonium-based fertilizers, which may have contributed to the numerical dominance of AOB.

A subsequent investigation of archaeal *amoA* gene abundance in the surface 0–10 cm of native remnant bushland adjacent to each of the agricultural trial sites and on the same soil type measured mean archaeal *amoA* gene abundances of 2.6 × 10^4^ and 1.4 × 10^4^ gene copies per g of soil at Buntine and Wongan Hills, respectively. This is approximately an order of magnitude higher than AOA abundance in the 0–10 cm depths of the agricultural soils, irrespective of treatment, potentially indicating a decline in AOA with agricultural management. A study of Scottish soils has previously provided evidence of a land use relationship with ammonia oxidiser communities with an increase in abundance of AOB in the agricultural ecosystems compared to the natural ecosystems surveyed, although AOA were always numerically more dominant^18^.

**Figure 2:**
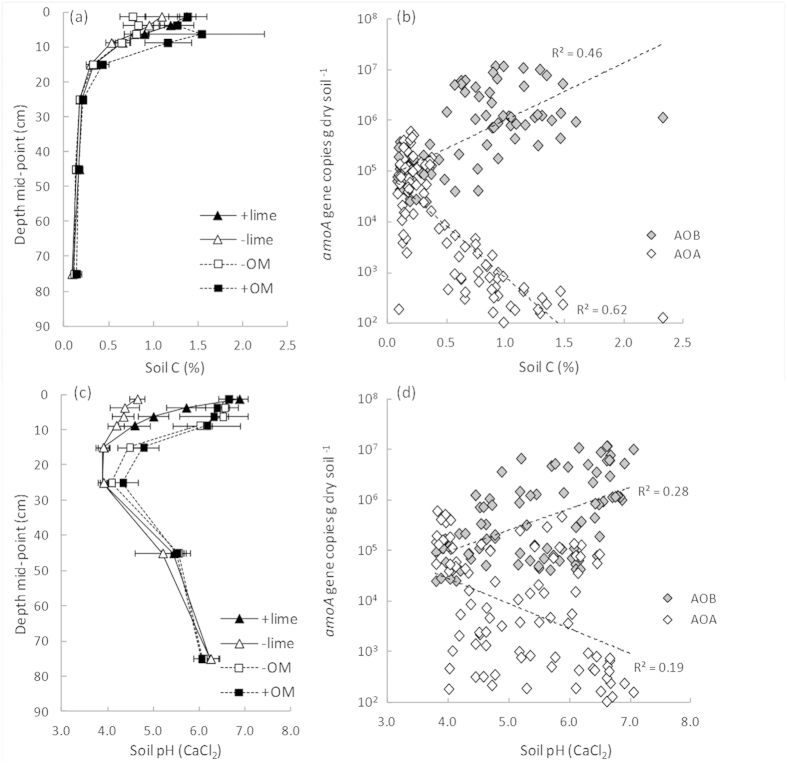
Depth profiles (0–90 cm) of soil carbon (**a**) and pH (**c**) in Western Australian agricultural soils collected from trial sites where soil pH (−lime versus +lime) or soil organic matter (−OM versus +OM) had been historically altered and the correlation of soil carbon and pH with bacterial and archaeal *amoA* gene abundance (**b** and **d**). Error bars represent ± 1 SE. Trendlines show a log-linear fit (all regressions significant at *P* < 0.001). Soil pH was determined in a 1:5 (w/w) soil suspension in 0.01 M CaCl_2_. Note: gene copy numbers are plotted on a log_10_ scale.

Examination of the relationship between changing AOB and AOA abundance with depth and two purported drivers of niche specialization between ammonia oxidisers, soil pH and substrate availability (as indicated by soil organic matter content), revealed significant positive correlations between these factors and AOB abundance and negative correlations with AOA abundance (Fig. 2). This suggests that AOA are better competitors in the more acidic, organic matter depleted soil conditions at depth which is in agreement with trends observed elsewhere^8^ and in physiological studies with the limited number of AOA in cultivation to date^16^. However, other variables such as water, carbon dioxide, oxygen and temperature, will also exhibit gradients with soil depth and may also play a role in regulating population abundances to depth.

In addition to the trends with depth this study also examined the effect of direct manipulation of soil pH through liming and indirect manipulation of substrate availability through organic amendment on ammonia oxidiser population abundance. The depth profiles demonstrate that, as expected, the influence of these agricultural practices was predominantly in the surface 10 cm (Fig. 2a,c). Increases in soil pH through liming (to near-neutral pH) did not alter total bacterial or archaeal abundances (*16S rRNA* gene copies), nor did it influence the uniformly low AOA abundance. However, the increased pH had a positive effect on AOB abundance in the surface 0–20 cm layer (Supplementary Fig. S1). This is in agreement with a study of acidic tea orchard soils in China which reported evidence of pH exerting a greater influence on AOB abundance than AOA abundance^31^.

**Figure 3:**
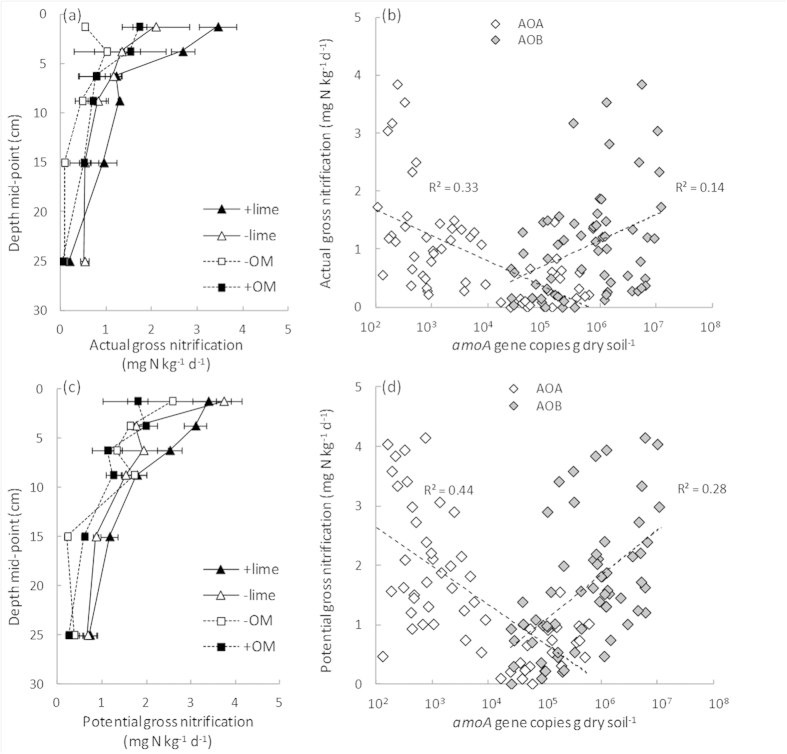
Depth profiles (0–30 cm) of actual (**a**) and potential (**c**) gross nitrification rates in Western Australian agricultural soils collected from trial sites where soil pH (−lime versus +lime) or soil organic matter (−OM versus +OM) had been historically altered and the correlation of nitrification rates with bacterial and archaeal *amoA* gene abundance (**b** and **d**). Error bars represent ± 1 SE. Trendlines show a log-linear fit (all regressions were significant at *P* < 0.001 for all except actual nitrification-logAOB where *P* = 0.003). Note: gene copy numbers are plotted on a log_10_ scale.

Increases in total organic carbon in response to the addition of extra plant residues (+OM treatment) were mirrored by increases in total nitrogen and nitrate and did not alter the soil pH. This suggests that, although the ammonium pool size remained low (<1 μg NH_4_^+^−N per g soil), the assumption that organic amendment increases substrate (ammonia) availability holds. The abundance of *16S rRNA* and *amoA* genes from both bacteria and archaea was found to decrease in the surface layer in the +OM treatment (Supplementary Fig. S1). This was surprising as the addition of extra organic carbon and nutrients was expected to increase the size of the prokaryotic community in general. However, actual gross nitrification rates were still higher in the surface (0–5 cm) soils of the +OM treatment (Fig. 3a), suggesting a more active ammonia-oxidising community. Although total carbon levels were the same in the sub-soil in the OM trial, all measured populations were generally more abundant below 15 cm in the +OM treatment. It is possible this was due to downward movement of dissolved and particulate organic matter. This pool is known to provide a major energy source for microorganisms and has been quantified to contribute as much as 42–49% of gross nitrification activity in a similar agricultural soil^32^.

In this study, ^15^N isotopic pool dilution was used to determine actual (no ammonium addition) and potential (with ammonium addition) nitrification rates at *in situ* pH values. Correlations between the abundance of ammonia oxidiser populations and potential nitrification rates have been observed previously^31^,^33^. However, analyses using potential nitrification assays^34^ are limited by the need to add substrate (ammonium) and the use of incubation conditions adjusted to a neutral pH which may inhibit species intolerant of those conditions^16^. Gross nitrification rates declined significantly with depth (*P* < 0.001) at both sites (Fig 3a,c) and increased significantly in response to liming (*P* < 0.001; actual nitrification rate and *P* < 0.05; potential nitrification rate). The addition of organic matter had no significant effect on gross nitrification rates (*P* = 0.109; actual nitrification rate and *P* = 0.365; potential nitrification rate). The abundance of AOB was found to positively correlate to both actual (*P* = 0.003) and potential (*P* < 0.001) gross nitrification rates while AOA abundance was negatively correlated (*P* < 0.001; Fig 3b,d). The negative correlation for AOA was a consequence of the increasing AOA abundance, but declining nitrification activity, with depth.-

We conclude that AOB are most likely responsible for the majority of soil nitrification activity which occurs in the surface of these semi-arid agricultural soils. While total archaeal abundance varied little with depth, it appears that most of the surface archaea did not contain *amoA* genes, although this is where the majority of nitrification activity takes place. While AOA may contribute to nitrification activity at depth, these findings highlight the distinct niche separation of AOA and AOB populations in these semi-arid agricultural soils with AOB populations dominant in the surface layer.

## Methods

### Soil collection

Soil was collected from two field trials on semi-arid agricultural soil within the central grain growing region of Western Australia: an organic matter trial at Buntine (30° 55’ S, 116° 21’ E) and a liming trial at Wongan Hills (30° 51’ S, 116° 44’ E). The region has a semi-arid climate, with hot, dry summers and cool, wet winters (when cropping occurs).

At Buntine mean annual rainfall is 285 mm, mean monthly temperatures range from 5.8–35.3 °C and actual temperatures range from −1.0–46.9 °C (calculated from 15 years of data, 1997–2014, Australian Bureau of Meteorology^35^). At the research site, soil temperatures (5 cm depth) ranged from 6–46 °C (2008–2012). Soil at the site is a deep sand (92% sand, 2% silt, 6% clay) and classified as a Basic Regolithic Yellow-Orthic Tenosol (Australian soil classification^36^), or a Haplic Arenosol (World Reference Base classification^37^). Soil organic matter (OM) treatments at Buntine were sampled from plots (10 m × 18 m) that were either tilled only (−OM) or tilled with the addition of extra plant residues (+OM) that had been surface applied at rate of 20 t ha^−1^ in 2003, 2006, 2010 and 2012 (12 days prior to sampling). This represented an additional 36 t ha^−1^, of which 7.0 t of C ha^−1^ was retained as extra soil organic carbon in the +OM treatment nine years after trial establishment (i.e. 64% more soil organic carbon in the +OM treatments compared to the −OM treatments). Tillage was by means of offset disks to 10 cm depth prior to seeding. Lime was applied to both treatments to maintain a surface pH > 5.5 to prevent sub-soil acidification in accordance with regional guidelines^38^.

At Wongan Hills, mean annual rainfall is 374 mm, with mean daily temperatures ranging from 11.7 °C–25.3 °C (calculated from 30 years of data, 1981–2010, Australian Bureau of Meteorology^35^). The soil at the experimental site is also a free-draining sand classified as an Acidic Ferric Yellow-Orthic Tenosol (Australian soil classification^36^). Soil pH treatments at Wongan Hills were sampled from plots (1 m × 4 m) that had either been limed (+lime; 3.5 t ha^−1^ in March 2009) or not limed (−lime). Three years after trial establishment the soil pH (CaCl_2_; 0–10 cm) was 4.4 in −lime and 5.5 in +lime treatments.

Soil was collected in March (summer) of 2012, prior to crop establishment. Three soil cores were collected from each replicate plot (n = 3) of each treatment. Soil from the three soil cores were combined to produce one sample per field plot at each of the following depth intervals (in cm): 0–2.5, 2.5–5, 5–7.5, 7.5–10, 10–20, 20–30, 30–60 and 60–90. Sub-samples for DNA extraction were frozen immediately upon collection in a portable freezer and transferred to −20 °C within 1 h.

### Nucleic acid extraction and qPCR

For each soil sample, DNA was extracted from duplicate 800 mg sub-samples using UltraClean™ DNA Isolation Kit (MoBio Laboratories Inc., Carlsbad, CA, USA). Cell lysis was performed using a Mini Bead beater (BioSpec products, Inc., USA) at 2500 rpm for 2 minutes. Duplicate DNA extractions were combined to give a total extract volume of 100 μl.

Functional genes, archaeal and bacterial *amoA* as well as archaeal and bacterial *16S rRNA* genes were quantified using a 7500FAST qPCR machine (Applied Biosystems, Life Technologies, USA). Each 20 μl qPCR reaction contained 10 μl of Power SYBR^®^ Green PCR Master Mix (Applied Biosystems), 0.2 μl of the specific forward and reverse primer at a concentration of 10 μM, 2 μL BSA (Ambion Ultrapure BSA; 5 mg ml^−1^), 2 μl of template DNA (8–115 ng) and 5.6 μL of water. DNA extracts were tested over a series of dilutions to determine if there was inhibition and the dilution which produced the highest copy number was used for further analysis. Primers and thermal cycling conditions for both bacterial (primers amoA-1F and amoA-2R) and archaeal (primers Arch-amoAF and Arch-amoAR) *amoA* genes were as described previously^13^. Archaeal *16S rRNA* gene primers Parch519F (CAGCMGCCGCGGTAA^39^) and Arch915R (GTGCTCCCCCGCCAATTCCT^40^) were used with the following thermal cycling conditions: 94 °C for 5 min then 40 cycles of 94 °C for 30 sec, 63 °C for 40 sec and 72 °C for 40 sec. Bacterial *16S rRNA* gene primers Eub338 (ACTCCTACGGGAGGCAGCAG^41^) and Eub518 (ATTACCGCGGCTGCTGG^42^) were used with the following thermal cycling conditions: 94 °C for 5 min then 40 cycles of 95 °C for 60 sec, 53 °C for 60 sec and 72 °C for 90 sec.

Melting curves were generated for each qPCR run and fluorescence data was collected at temperatures above the Tm of the primers but below that of the target (78 °C for both *amoA* genes, 72 °C for archaeal and 75 °C for bacterial *16S rRNA* genes) to verify product specificity. Each qPCR reaction was run in triplicate. Standard curves were generated using dilutions of linearized cloned plasmids. Template amplified with each primer pair described above, was cloned with the P-GEM T-easy system (Promega, USA), plasmid DNA extracted and inserts sequenced using Big Dye Terminator chemistry (Australian Genome Research Facility, Western Australia) to confirm correct length and identity. The standard curve gene sequences were as described previously^13^. Standard curves generated in each reaction were linear over four orders of magnitude (10^4^ to 10^7^ gene copies) with r^2^ values greater than 0.99. Efficiencies for all quantification reactions were 80–100%.

### Gross nitrification

Gross nitrification rates were determined by ^15^N isotopic pool dilution (see Murphy *et al.*^43^ for theory and methodological considerations) in soil adjusted to 45% water filled pore space and incubated at 25 °C. Subsamples of soil (20 g dry weight equivalent) were packed into 120 ml vials at a bulk density of 1.4 g cm^−3^. To determine actual nitrification rates, 1 ml of ^15^N enriched (60 atom % excess) KNO_3_ was applied as multiple droplets to the vials to obtain a concentration of 5 μg N g^−1^ soil. Potential nitrification rates were determined in separate vials by adding 1 ml of ^15^N enriched (60 atom % excess) KNO_3_ (5 μg N g^−1^ soil) and (NH_4_)_2_SO_4_ at natural abundance (5 μg N g^−1^ soil). The vials were incubated with the lids closed to avoid water loss and aerated every 24 h. Extractions occurred 2 h and 96 h after ^15^N addition with 80 ml of 0.5 M K_2_SO_4_ for 1 h on an end-over-end shaker, allowed to settle for 30 min and then filtered through Whatman No. 42 filter paper. Soil extracts were prepared for ^15^N/^14^N isotope ratio analysis using a modified diffusion method^44^,^45^ with subsequent isotope ratio analysis (SERCON 20–22 mass spectrometer connected with an Automated Nitrogen Carbon Analyzer; Sercon, UK). Gross nitrification was calculated using the equation by Kirkham and Bartholomew^46^.

### Statistical analyses

All data were statistically analyzed using mixed model Restricted Maximum Likelihood (REML) repeated measures analysis using GenStat v14.0^47^. Skewed data was corrected by transforming to the natural logarithm prior to analysis. AOB data could not be normalized by natural log transformation so was transformed by log_10_ prior to analysis. A significance level of 5% was used for all analysis and the Power model (City block metric) was used allowing for variance heterogeneity.

## Additional Information

**How to cite this article**: Banning, N. C. *et al.* Ammonia-oxidising bacteria not archaea dominate nitrification activity in semi-arid agricultural soil. *Sci. Rep.* **5**, 11146; doi: 10.1038/srep11146 (2015).

## Supplementary Material

Supplementary Information
