# Supplementary material for: Ammonia-oxidising bacteria not archaea dominate nitrification activity in semi-arid agricultural soil
Source: Sci Rep. 2015 Jun 8;5:11146. doi: 10.1038/srep11146 (PMC4459192; doi:10.1038/srep11146)
Supplement: Supplementary Information [file srep11146-s1.pdf]

Ammonia oxidising bacteria not archaea dominate nitrification activity in semi-arid agricultural soil

Natasha C. Banning, Linda D. Maccarone, Louise M. Fisk and Daniel V. Murphy*

Soil Biology and Molecular Ecology Group, School of Earth and Environment, Institute of Agriculture, The University of Western Australia, Crawley, WA 6009, Australia.

* Corresponding author: daniel.murphy@uwa.edu.au

Supplementary Information

Supplementary Fig. S1. Abundance of bacterial *16S rRNA* genes (a,f), bacterial *amoA* genes (b,g), archaeal *16S rRNA* genes (c,h) and archaeal *amoA* genes (d,i) in soil cores (0-90 cm depth) from two Western Australian agricultural field trials with treatments + or – organic matter (OM; top row) and + or – lime (bottom row). Treatment effects on soil C (e) and soil pH (j) are shown for the OM and liming trial sites, respectively. Note: gene copy numbers are plotted on a $\log_{10}$ scale.

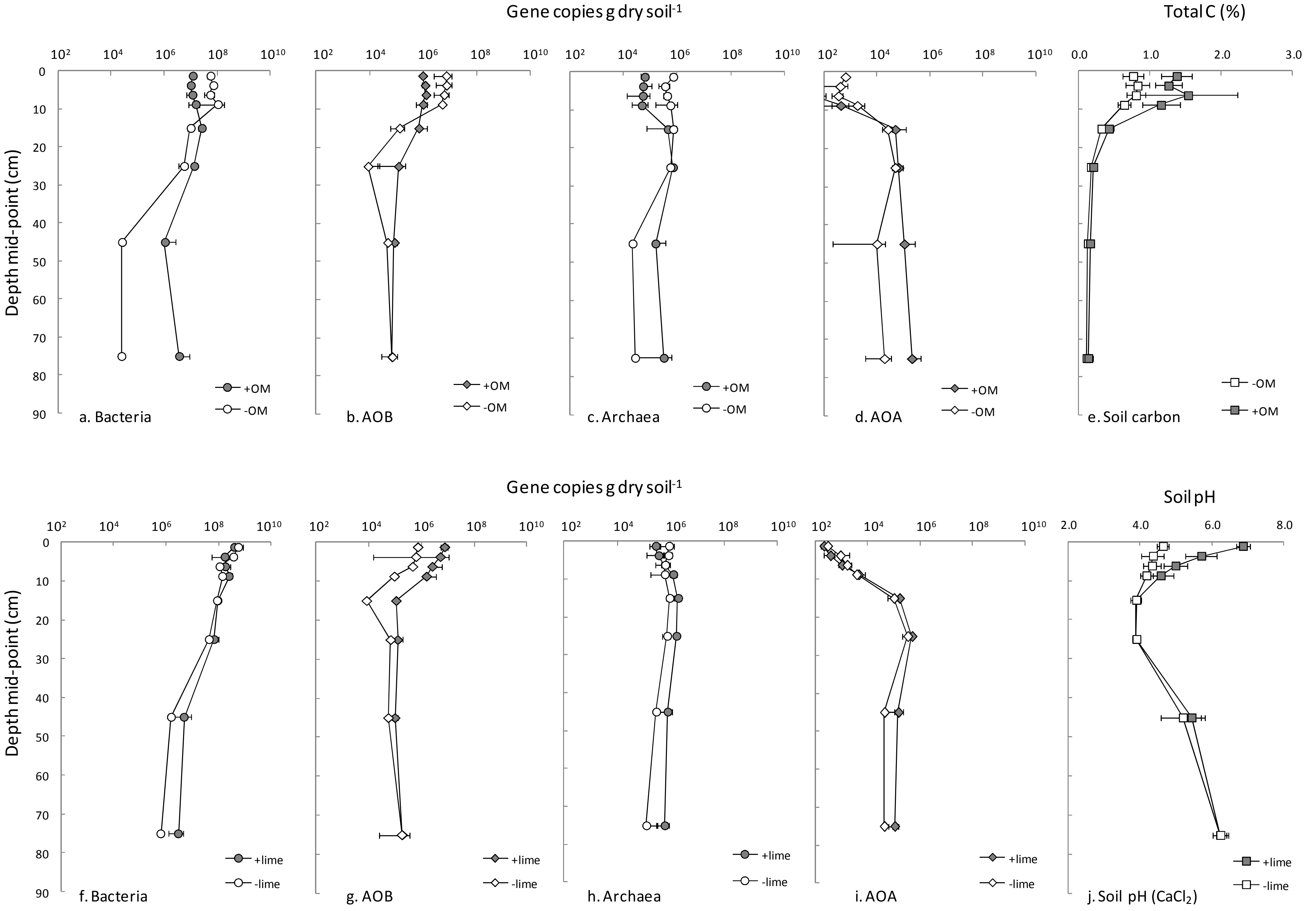
Gene copies g dry soil-1
Total C (%)
Depth mid-point (cm)
+OM
-OM
a. Bacteria
b. AOB
c. Archaea
d. AOA
e. Soil carbon
Gene copies g dry soil-1
Soil pH
+lime
-lime
f. Bacteria
g. AOB
h. Archaea
i. AOA
j. Soil pH (CaCl2)
